# Comparative Transcriptome Analysis of Mink (*Neovison vison)* Skin Reveals the Key Genes Involved in the Melanogenesis of Black and White Coat Colour

**DOI:** 10.1038/s41598-017-12754-0

**Published:** 2017-09-29

**Authors:** Xingchao Song, Chao Xu, Zongyue Liu, Zhigang Yue, Linling Liu, Tongao Yang, Bo Cong, Fuhe Yang

**Affiliations:** Key Laboratory of Special Economic Animal Genetic Breeding and Reproduction, Ministry of Agriculture, State Key Laboratory for Molecular Biology of Special Economic Animals, Institute of Special Economic Animal and Plant Sciences, Chinese Academy of Agricultural Sciences, Changchun, 130112 China

## Abstract

Farmed mink (*Neovison vison*) is one of the most important fur-bearing species worldwide, and coat colour is a crucial qualitative characteristic that contributes to the economic value of the fur. To identify additional genes that may play important roles in coat colour regulation, Illumina/Solexa high-throughput sequencing technology was used to catalogue the global gene expression profiles in mink skin with two different coat colours (black and white). RNA-seq analysis indicated that a total of 12,557 genes were differentially expressed in black versus white minks, with 3,530 genes up-regulated and 9,027 genes down-regulated in black minks. Significant differences were not observed in the expression of MC1R and TYR between the two different coat colours, and the expression of ASIP was not detected in the mink skin of either coat colour. The expression levels of KITLG, LEF1, DCT, TYRP1, PMEL, Myo5a, Rab27a and SLC7A11 were validated by qRT-PCR, and the results were consistent with RNA-seq analysis. This study provides several candidate genes that may be associated with the development of two coat colours in mink skin. These results will expand our understanding of the complex molecular mechanisms underlying skin physiology and melanogenesis in mink and will provide a foundation for future studies.

## Introduction

Farmed mink (*Neovison vison*), which belongs to the *Mustelidae* family in the order *Carnivora*, is one of the most important fur-bearing species worldwide, and coat colour is a crucial qualitative characteristic that contributes to the economic value of the fur. Wild minks as well as black and dark-brown farmed minks, which are referred to as standard minks, are normally uniformly coloured over the entire body^1^. Regarding the quantitative characteristics of standard minks, the coat colour types are often a common breeding objective. At least 31 different genes that control colour types in standard minks have been identified, including dominant and recessive genes. Factors that determine coat colour in minks are becoming increasingly important. White coats hold the greatest economic value because of their ability to be dyed to virtually any colour, whereas interest in natural colours is increasing because of the green revolution and consumer preference for natural products.

In numerous vertebrates, the colour of hair, feathers and skin is primarily determined by the amount and distribution of two pigments, eumelanin (black or brown) and pheomelanin (red or yellow), which are secreted by mature melanocytes at the base of the epithelium^2^. In mice, more than 170 genes that affect pigmentation in different ways have been cloned, and 207 other coat colour-associated genetic loci have been detected but not cloned^3^. However, the molecular genetic mechanisms underlying coat colour regulation in minks are not fully understood because few candidate genes potentially involved in pigmentation have been identified except for the MLPH, LYST, tyrosinase (TYR), MITF and tyrosinase-related protein 1 (TYRP1) genes^4–8^. Despite considerable knowledge of the genetic regulation of coat colour in mice^9–11^, cows^12,13^, goats^14,15^, sheep^16,17^ and pigs^18–20^, the molecular and cellular mechanisms regulating coat colour in fur-bearing species, such as mink, are not fully understood. This information is critical for enhancing our basic understanding of the regulation of melanogenesis and for identifying novel pharmacological and molecular genetics approaches to regulate or select for coat colour in fur-bearing species.

**Figure 1:**
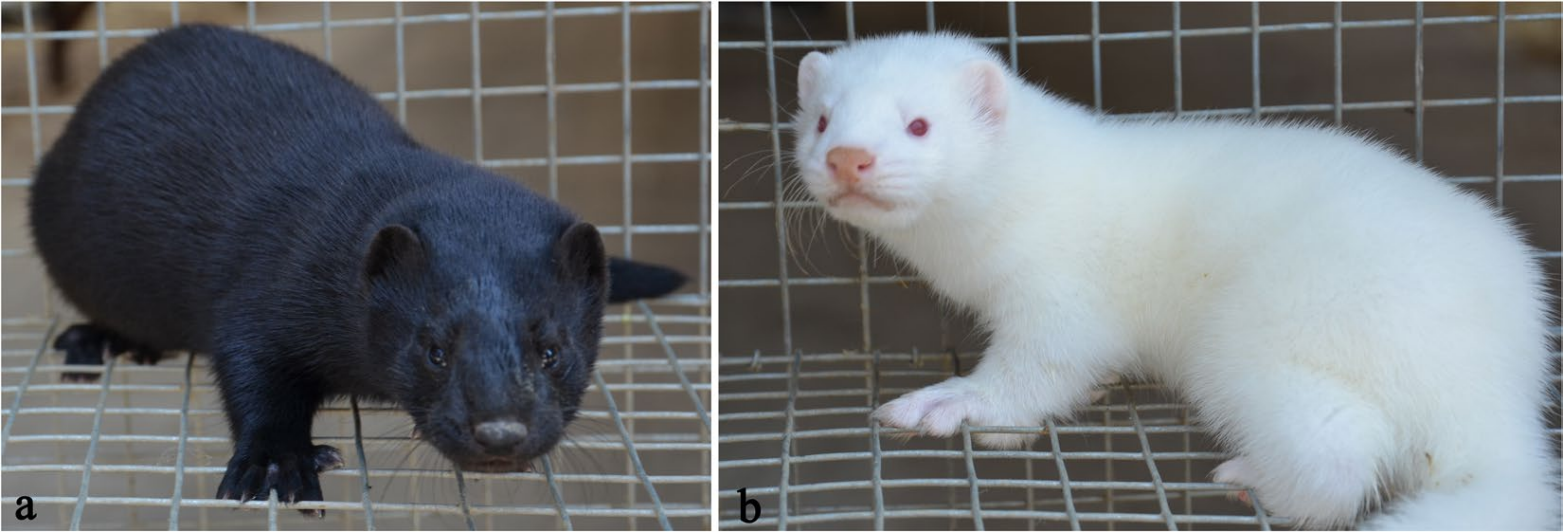
Two coat colour phenotypes of mink representing 2 Chinese cultivated breeds, (**a**) BLM_S: Jinzhou black mink, black coat colour over the entire body with dark brown nose and black eyes. (**b**) WHM_S: Jilin white mink, white coat colour, where the red eyes can noticed, used in this study.

Transcriptional profiling is a powerful approach for the global identification of genes and their functional expression in various tissues^21,22^, including skin^23,24^, hair follicles^25^ and feather bulbs^26^. Limited information is currently available on coat colour-associated differences in the transcriptome profiles of skin in fur-bearing species. To investigate genes that play important roles in coat colour regulation in mink skin and gain insights into the molecular mechanisms responsible for the biochemistry of the skin and fibres (including the pigmentation) of the mink, we investigated the transcriptome profiles of the skin of minks with wild-type and white coat colours (Fig. 1) using high-throughput deep RNA sequencing. The results provided novel insights into the differences in gene expression associated with coat colour and identified key genes implicated in the melanogenesis pathway. These findings will enable a better understanding of the molecular mechanisms involved in skin pigmentation and provide a valuable theoretical basis for the selection of natural colour traits.

## Results

### Illumina paired-end sequencing and *de novo* assembly

To determine the skin transcriptomes of minks, six sequencing libraries were prepared from skin with black and white coat colours and sequenced using the Illumina paired-end technique. In total, 173,485,532 and 183,877,224 raw reads were generated from the skin with black and white coat colours, respectively, and yielded 21.28 and 22.52G clean bases, respectively (Table 1). The combined numbers of Illumina read pairs and clean bases for the two types of mink skin were 350,424,598 and 43.80G. Of the raw reads from black mink, more than 93.63% of the bases had a Q value > 20 (an error probability of 0.045%), and for white mink, 94.05% had a Q value > 20 (an error probability of 0.042%). The GC-contents were 49.60% and 49.79% for black and white mink skin, respectively. After removing adaptors and low-quality data, 170,278,748 and 180,145,850 clean reads were obtained from the black and white mink skin, respectively. The high-quality reads were then used for *de novo* assembly using Trinity. Using overlapping information in the high-quality reads, Trinity generated 491,191 transcripts with an average length of 1,005 bp and an N50 of 2,644 bp. The sequence length distribution of the transcripts is shown in Table 2. Further assembly analysis showed that all transcripts contributed to 403,725 unigenes, with an average length of 623 bp and an N50 of 917 bp.

**Table 1:** Summary of the sequence assembly after Illumina deep sequencing.

Sample name	Raw Reads	Clean reads	Clean bases	Error rate (%)	Q20 (%)	Q30 (%)	GC (%)
BLM_S1-L	29,731,089	29,186,255	3.65G	0.04	94.62	89.61	49.70
BLM_S1-R	29,731,089	29,186,255	3.65G	0.05	92.65	86.93	49.74
BLM_S2-L	29,899,925	29,305,424	3.66G	0.04	94.72	89.82	49.25
BLM_S2-R	29,899,925	29,305,424	3.66G	0.05	92.75	87.11	49.31
BLM_S3-L	27,111,752	26,647,695	3.33G	0.04	94.63	89.63	49.78
BLM_S3-R	27,111,752	26,647,695	3.33G	0.05	92.29	86.41	49.82
WHM_S1-L	28,796,205	28,215,855	3.53G	0.04	94.95	90.21	49.80
WHM_S1-R	28,796,205	28,215,855	3.53G	0.04	93.28	87.96	49.86
WHM_S2-L	28,913,848	28,344,875	3.54G	0.04	94.98	90.25	49.66
WHM_S2-R	28,913,848	28,344,875	3.54G	0.04	93.59	88.45	49.70
WHM_S3-L	34,228,559	33,512,195	4.19G	0.04	94.74	89.83	49.83
WHM_S3-R	34,228,559	33,512,195	4.19G	0.05	92.73	87.05	49.89
BLM_S-Summary	173,485,532	170,278,748	21.28G	0.045	93.63	88.25	49.60
WHM_S-Summary	183,877,224	180,145,850	22.52G	0.042	94.05	88.96	49.79
Summary	357,362,756	350,424,598	43.80G				49.70

L: Reads sequencing from the left; R: Reads sequencing from the right; Q20: The percentage of bases with a Phred value > 20; Q30: The percentage of bases with a Phred value > 30.

**Table 2:** Summary of the mink skin transcriptome.

Category	Number of length interval				Total number	Mean length (bp)	Max length (bp)	N50 (bp)	Total nucleotides
	200–500 bp	500-1 kbp	1 k–2 kbp	>2 kbp					
Transcripts	299,148	79,767	47,179	65,097	491,191	1,005	27,550	2644	493,857,304
Unigenes	287,584	67,497	28,377	20,267	403,725	623	27,550	917	251,538,954

### Gene functional annotation of all non-redundant unigenes

Unigene annotation was performed via a BLAST search with an E-value threshold of 1e-5 against the Nr (NCBI non-redundant protein sequences), Nt (NCBI nucleotide sequences), COG (Clusters of Orthologous Groups), PFAM (Protein family), Swiss-Prot, Kyoto Encyclopaedia of Genes and Genomes (KEGG) and Gene Ontology (GO) databases. Among the 403,725 assembled unigenes, 47,906 (11.86%) were found to have significant BLAST results in the Nr database. A total of 37,803 unigenes (9.36%) were annotated with the Swiss-Prot databases. The number of unigenes with significant similarity to sequences in the COG, KEGG, PFAM and Nt databases were 14,445 (3.57%), 18,839 (4.66%), 41,110 (10.18%) and 116,498 (28.85%), respectively (Table 3).

**Table 3:** Gene annotation by searching against public databases.

Public database	Number of unigenes	Percentage (%)
Annotated in Nr	47,906	11.86
Annotated in Nt	116,498	28.85
Annotated in KO	18,839	4.66
Annotated in Swiss-Prot	37,803	9.36
Annotated in PFAM	41,110	10.18
Annotated in GO	41,522	10.28
Annotated in COG	14,445	3.57
Annotated in all databases	8,712	2.15
Annotated in at least one database	135,468	33.55
Total unigenes	403,725	100

### Functional classification by GO, COG and KEGG

**Figure 2:**
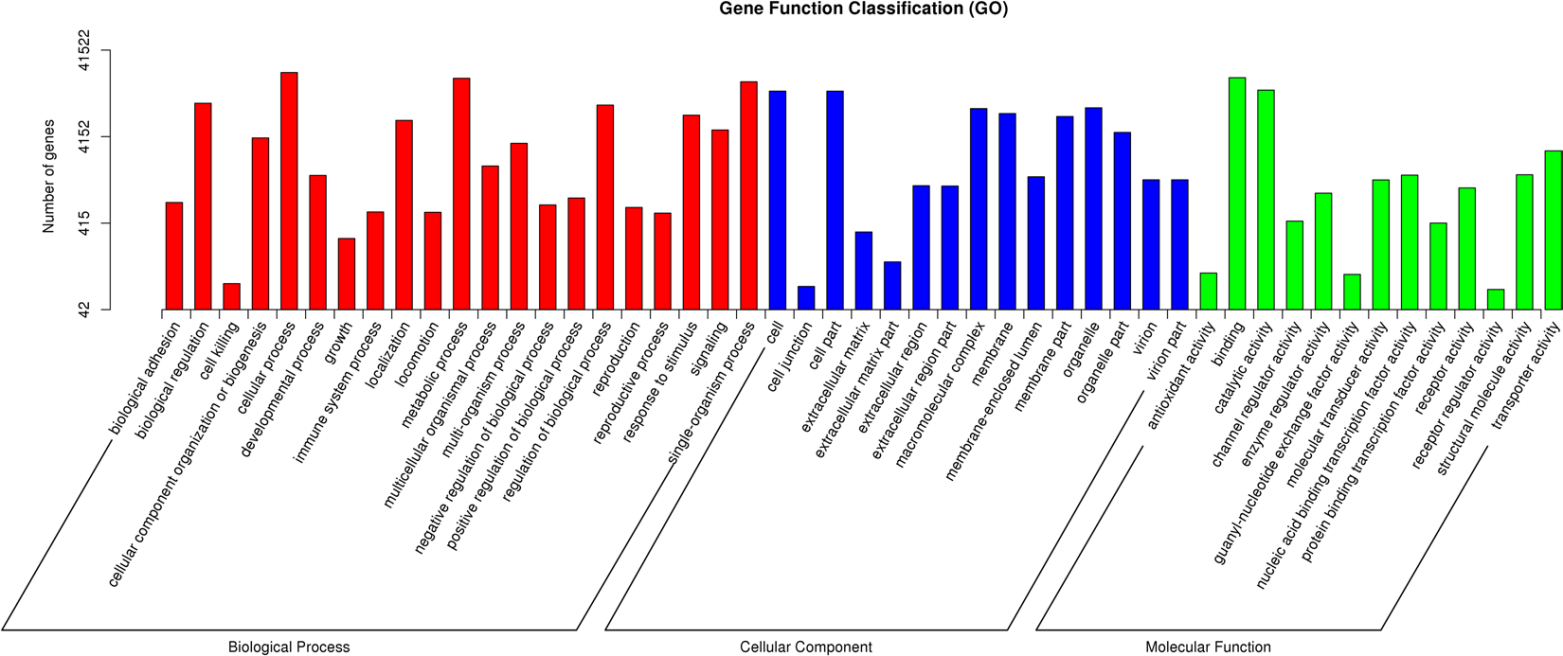
Function classification of the assembled unigenes based on Gene Ontology (GO) categorization. The 41,522 unigenes were summarized in three main GO categories: biological processes (BP), cellular components (CC) and molecular functions (MF). The X axis indicates the next level of term in the three major categories of GO, and the Y axis indicates the number of unigenes annotated to the term.

To classify the functions of the predicted mink unigenes, a GO analysis, which is an internationally standardized gene functional classification system, was performed. In total, 41,522 unigenes with BLAST matches to known proteins were classified into 3 functional categories: Biological process (GO: 0008150), Cellular component (GO: 0005575) and Molecular function (GO: 0003674) (Fig. 2). For the biological process terms, these unique sequences were grouped into 21 classifications, and most were ‘cellular process’, ‘metabolic process’ and ‘single-organism process’. Cell, cell part and organelle were the most represented categories in cellular components. The most common molecular functions were binding, catalytic activity and transporter activity (Fig. 2).

**Figure 3:**
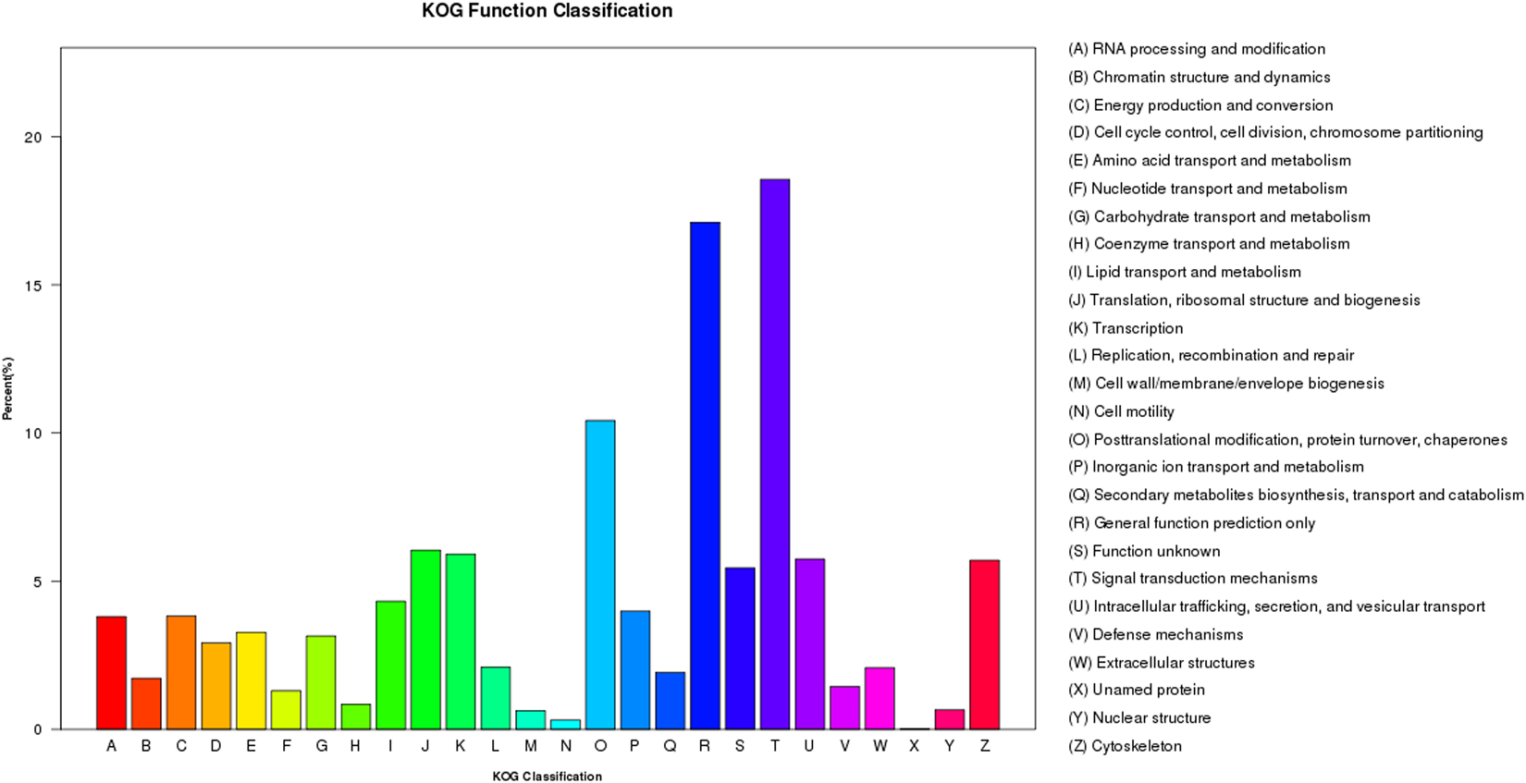
Histogram of Clusters of eukaryotic Orthologous Groups (COG) classification. The 14,445 unigenes were aligned to the COG database to predict and classify possible functions. The X axis indicates the name of 26 group in COG categories, and the Y axis indicates the percentage of the total number of annotated genes.

COG is a classification system based on orthologous genes, which have the same function and a common ancestor. In total, 14,445 unigenes were annotated to 26 groups with the COG database (Fig. 3). The (T) signal transduction mechanism category, with 2,680 annotated unigenes, was the largest category. The second category, (R) General function, contained 2,471 unigenes. (O) Posttranslational modification, protein turnover and chaperones was the third most common category and contained 1,505 unigenes. (X) Unnamed protein contained the fewest unigenes with 3 (Fig. 3).

**Figure 4:**
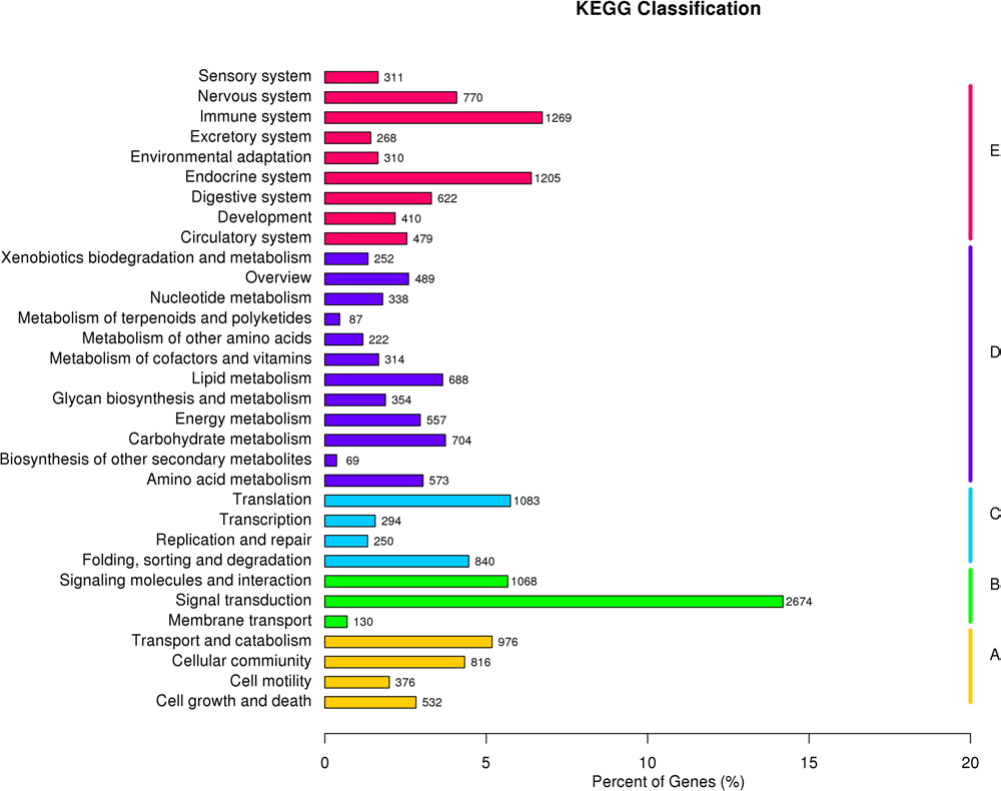
Pathway assignment based on the Kyoto Encyclopedia of Genes and Genomes (KEGG) database. 19,330 unigenes were assigned into 5 KEGG biochemical pathways. (**A)**, Cellular Processes (CP); (**B**), Enviromental Information Processing (EIP); (**C**), Genetic Information Processing (GIP); (**D**), Metabolism (M); (**E**), Organismal Systems (OS).

The assembled unigenes were assigned to the biochemical pathways described in KEGG (Fig. 4). The KEGG database contains a systematic analysis of inner-cell metabolic pathways and gene product functions. Pathway-based analyses help to further determine the biological functions of genes. In total, 19,330 unigenes were assigned to 5 KEGG biochemical pathways: Organismal Systems (5,644 unigenes), Metabolism Pathway (4,647), Environmental Information Processing (3,872), Cellular Processes (2,700) and Genetic Information Processing (2,467). The largest group, Organismal Systems, was well represented among the 5,644 mink unigenes, with the most genes associated with the immune system (1,269), the endocrine system (1,205), the nervous system (770), the digestive system (622), the circulatory system (479), development (410), the sensory system (311), environmental adaptation (310) and the excretory system (268). Pathways related to metabolic pathways were the second most common, including genes involved in carbohydrate metabolism (704), lipid metabolism (688), amino acid metabolism (573), energy metabolism (557), glycan biosynthesis and metabolism (354) and metabolism of cofactors and vitamins (314). The third largest group consisted of environmental information processing, with a majority of the proteins involved in signal transduction (2,674), signalling molecules and interaction (1,068) and membrane transport (130). Pathways related to cellular processes and genetic information processing were also well represented by mink unigenes. These results provide a valuable resource for investigating the metabolic pathways in the skin of minks with different coat colours.

### Differentially expressed genes (DEGs) in mink skin with black and white coat colours

**Figure 5:**
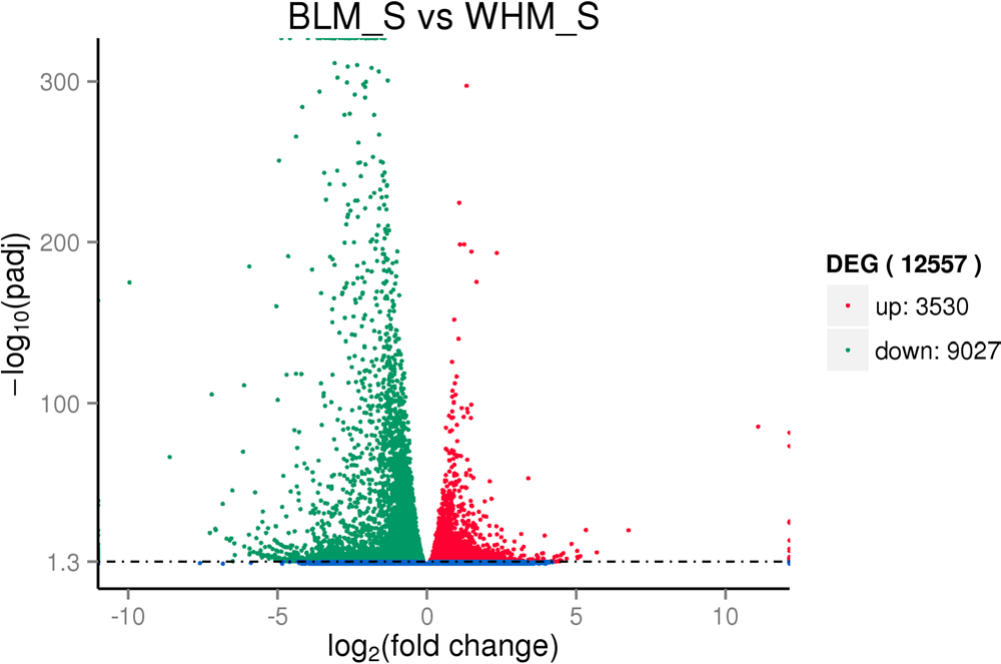
Comparison of expression patterns of differential unigenes identified between two mink skins with black and white coat colour. The X axis indicates gene expression changes in different samples, and the Y axis indicates the significant degree of gene expression changes. Scattered points represent each gene, the red dots represent differentially up-regulated genes, the green dots represent differentially down-regulated genes, and the blue dots represent no significant difference gene. In total, 12,557 unigenes were identified as differentially expressed between skins with two coat colours, including 3,530 genes that were up-regulated and 9,027 down-regulated genes.

**Figure 6:**
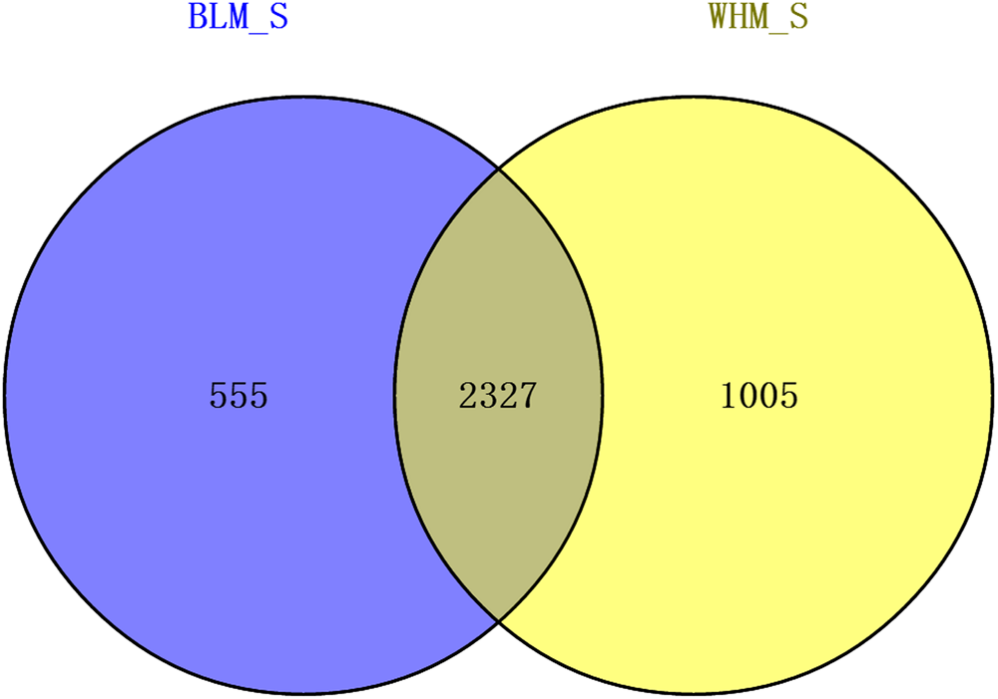
The differentially expressed genes (DEGs) that are unique or shared between BLM_S and WHM_S. BLM_S refers to the black coat colour group, and WHM_S refers to the white coat colour group. The numbers in each section of the figure indicate the number of DEGs in the indicated comparison (|log_2_Foldchange| > 1 and padj < 0.05).

M vs A plots can be used to determine any systematic bias that may be present between conditions (A: log_2_Foldchange; M: −log_10_padj). Therefore, we used the log_2_Foldchange value combined with -log_10_padj to assess every gene expressed between BLM_S and WHM_S. Volcano plots exploring the relationship between the fold change and the significance are shown in Fig. 5. We identified 12,557 unigenes as DEGs, including 3,530 up-regulated and 9,027 down-regulated unigenes. To better investigate the biological mechanism of melanogenesis, it is important to identify the DEGs between the two mink skins with different coat colour. A gene expression comparison showed that a total of 3,887 unigenes were differentially expressed between BLM_S and WHM_S when |log_2_Foldchange| > 1 and padj < 0.05 were used as the cut-off values (Fig. 6). Of these, 2,327 unigenes were significantly differentially expressed in both BLM_S and WHM_S. Moreover, 555 and 1,005 unigenes were uniquely expressed in BLM_S and WHM_S, respectively. All the DEGs are illustrated in Fig. 6. In mice, approximately 171 genes that affect pigmentation in different pathways have been cloned and identified, and another 207 coat colour-associated genetic loci have been detected but not yet cloned. Those known coat colour genes are routinely classified into five general functions: Melanocyte development, Components of melanosomes and their precursors, Melanosome construction/protein routing, Melanosome transport and Eumelanin and Pheomelanin. The expression levels of 19 of the aforementioned coat colour genes were detected in mink skin in the present study, with 7 genes showing higher expression in black mink skin and 12 genes showing higher expression in white mink skin (Table 4). The coat colour genes in the ‘Eumelanin and Pheomelanin’ functional category showed higher expression in white mink skin. Among the coat colour genes showing higher expression in black mink skin, the TYRP1 gene showed the highest expression in black mink skin versus white mink skin, and it was followed by the Pax3, dopachrome tautomerase (DCT), Sox10, LYST, KITLG and Rab27a genes. Interestingly, in white mink skin, the highest expression was for the Oca2 gene, which is associated with oculocutaneous albinism type 2 (Oca2), a pink-eyed dilution (*p*) locus.

**Table 4:** Differentially expressed known coat colour genes in black vs white mink skin.

Classification	Gene name	Unigene ID	Relative expression	log_2_Foldchange	p-adjusted	Significant
Melanocyte development	KITLG	c87266_g2	Up-regulation	0.5004	3.57E-08	True
	LEF1	c89232_g1	Down-regulation	−0.95058	2.20E-06	True
	MITF	c88366_g1	Down-regulation	−0.19341	0.024184	True
	Sox10	c93414_g9	Up-regulation	0.91883	4.30E-31	True
	Arcn1	c92006_g1	Down-regulation	−0.3903	4.39E-20	True
	Edn3	c78084_g1	Down-regulation	−1.4423	0.029303	True
	Pax3	c91778_g4	Up-regulation	1.0672	0.047137	True
Components of melanosomes and their precursors	DCT	c85800_g1	Up-regulation	1.0592	1.11E-08	True
	TYRP1	c82939_g1	Up-regulation	2.9322	1.39E-14	True
	PMEL	c82046_g1	Down-regulation	−1.4447	4.37E-67	True
Melanosome construction/protein routing	LYST	c87901_g1	Up-regulation	0.54767	2.59E-24	True
	Oca2	c82300_g1	Down-regulation	−1.4642	4.74E-08	True
	Rab38	c76882_g1	Down-regulation	−0.24669	0.001429	True
Melanosome transport	MLPH	c92573_g2	Down-regulation	−0.42379	0.00265	True
	Myo5a	c92365_g1	Down-regulation	−0.16804	0.020396	True
	Myo7a	c93880_g1	Down-regulation	−0.91998	7.93E-22	True
	Rab27a	c84818_g1	Up-regulation	0.41901	0.000107	True
Eumelanin and Pheomelanin	Ostm1	c88620_g1	Down-regulation	−0.68955	3.91E-18	True
	Sox18	c93414_g7	Down-regulation	−0.81495	3.99E-08	True

### Validation of the differentially expressed mRNAs in mink skin

**Figure 7:**
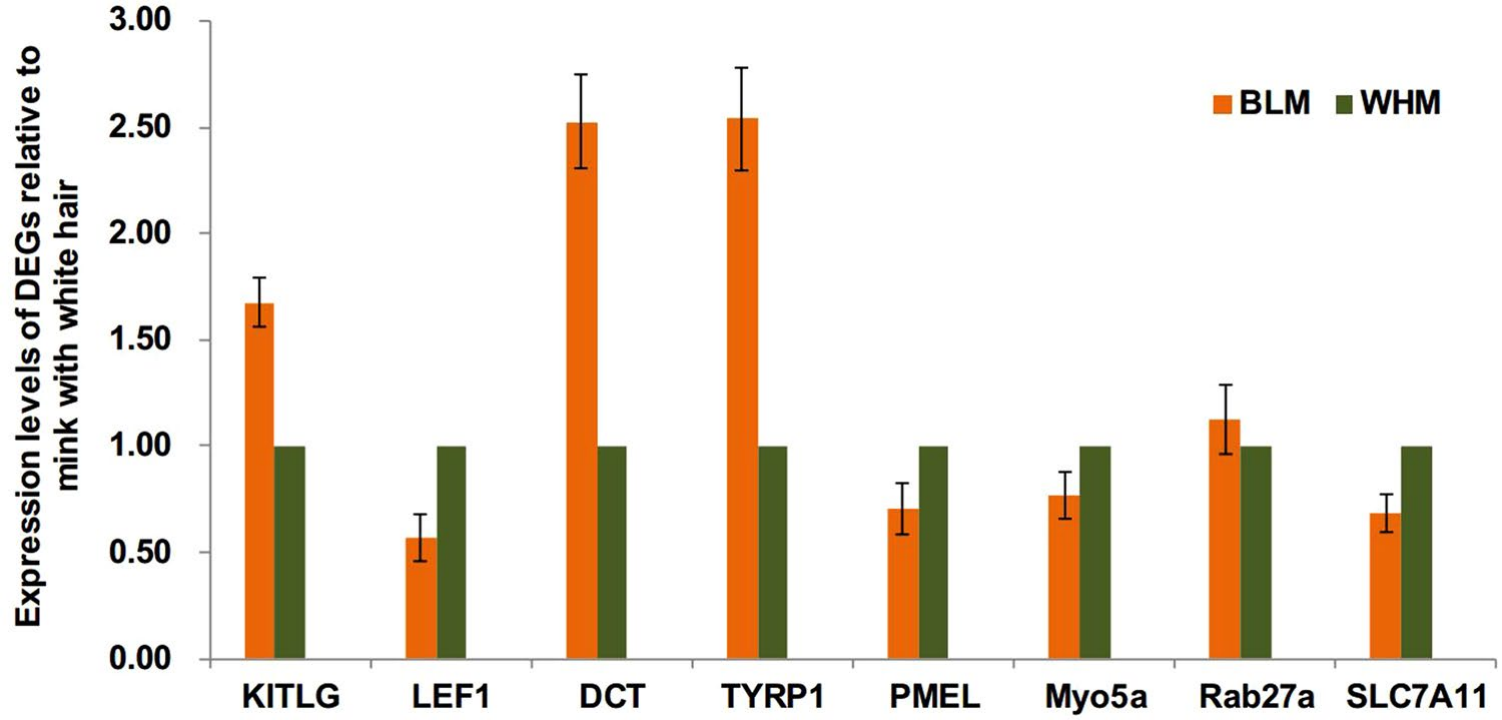
Quantitative real time PCR (qRT-PCR) validation of DEGs in mink skin with two different coat colours. Abundance of target genes was normalized relative to abundance of β-actin gene. Bars in each panel represent the mean ± standard (n = 3).

We further validated eight DEGs using qRT-PCR with gene-specific primers to confirm the gene expression patterns. These eight genes were designated as likely to be involved in melanogenesis or the transcriptional regulation of the melanogenesis pathway based on previous publications or best blast matches to ferret. The qRT-PCR gene expression patterns were compared with the data obtained from the comparative transcriptome analysis. The results showed that there was a strong correlation between the RNA-seq data and qRT-PCR results (Fig. 7).

## Discussion

Mammalian coat colour exhibits a wide range of shades and is controlled by melanin production in melanocytes (melanogenesis). Melanogenesis involves intricate molecular regulation^27^, and a number of recent publications have summarized our current understanding of the process of melanin production in skin and coats^28–30^. To clarify the complicated molecular mechanisms of coat colour formation, previous studies have reported the global gene expression profiles in the skin of chickens^31^, sheep^32^ and common carp^33,34^ using Illumina sequencing technology. Another study that compared the genomic sequences available from BAC clones^35^ with mink transcript sequences via HiSeq. 2000 Illumina pair-end sequencing^36^ revealed seven single nucleotide polymorphisms (SNPs) in coat colour genes. A number of studies, including GWAS (genome-wide association studies), have identified many polymorphisms regulating human facial pigmented spots, hair and eye colour, such as MC1R, ASIP, IRF4, BNC2, OCA2, TYR, TYRP1, KITLG, SLC24A5, SLC45A2 and EDNRB^37–39^. To further investigate the genes that may play important roles in mink skin, particularly in coat pigmentation, over 350 million clean paired-end transcriptome reads were generated from mink skin with black and white coat colours using an Illumina HiSeq. 2500 system. Our study offers new information related to the gene expression profiles for mink skin with two different coat colours. From these clean reads, 403,725 known unigenes were identified as expressed in mink skin, and 12,557 were differentially expressed in black versus white mink skin. Although the study design was not optimal because single pooled samples (n = 3 per coat colour) were used in transcriptome sequencing analysis, with the same three samples from white and black mink skin used individually for quantitative real-time PCR validation of sequencing results, which led to limited biological replication, the results of this experiment have significantly enhanced our understanding of the composition of the mink skin transcriptome and the potential differences in gene expression associated with coat colour, which will provide a foundation for future studies.

The GO and KEGG pathway analyses of DEGs revealed that most were associated with the metabolic process (MP), binding (B) and catalytic activity (CA) ontology categories. Of particular interest in our dataset were pathways related to catabolic, metabolic and biosynthetic pigment processes. Of the DEGs, the genes in the categories related to ‘the components of melanosomes and their precursors’ and ‘melanocyte development’, including the genes TYRP1, DCT and KITLG, were up-regulated in the skin from minks with black coat colour. The function of genes in ‘the components of melanosomes and their precursors’ and ‘melanocyte development’ categories are melanin synthesis and differentiation of relevant cells^3^. The darker pigmentation of hair and skin is associated with a higher number of melanosomes, although the number of melanocytes remains constant^40^. Melanocytes in black hair follicles contain the highest number of melanosomes, whereas fewer melanosomes are found in white hair bulbs^41^. The relationship of less melanin with the lighter hair phenotype has been identified in several species, including mice^28,42,43^, horses^44^, sheep^45^, alpacas^46^ and humans^43^. In this study, these results provide strong evidence that there is a significant difference in the level of pigmentation and melanogenesis between black and white coat colours of mink skin. However, further investigation is still needed to confirm the regulatory relationships of these genes.

The colour of skin, coat, feathers, and shells in mammals, birds, and bivalve molluscs is determined mainly by two melanins: eumelanin and pheomelanin^47–49^. For melanogenesis, TYR, TYRP1, and dopachrome tautomerase (DCT) were directly involved in the synthesis of melanins, and TYRP1 and DCT are involved in the distal eumelanic pathway and are active in the eumelanogenic pathway. In this study, Illumina HiSeq. 2500 pair-end sequencing indicated high expression of TYRP1 and DCT in mink with a black coat colour from skin with the pure black phenotype. These results demonstrated that a lack of TYRP1 and DCT expression led to a deficiency in the biosynthesis of melanin in mink skin with the white coat colour phenotype and may be the direct cause of white coat colour formation. This result is similar to observations in the American mink with the palomino phenotype, which is caused by a large insertion in intron 2 of the TYRP1 gene^8^, which is consistent with studies in domestic sheep, where the TYRP1 and DCT genes showed higher expression in black sheep skin^32^. KITLG, also known as stem cell factor (SCF) and mast cell growth factor, binds to and activates KIT and plays a crucial role in the development and maintenance of the melanocyte and melanin synthesis in fish, birds and mammals^50,51^, although its expression varies among species. Our RNA-seq and qRT-PCR results all showed that the TYRP1, DCT and KITLG genes were significantly up-regulated in the skin of mink with black hair, indicating that these genes may be responsible for black hair pigmentation in mink. However, the details of the interactions of the regulatory pathways in mink skin remain to be further investigated.

The premelanosome protein (PMEL, also known as SILV or PMEL17), a key component of mammalian melanosome biogenesis, is required for the generation of cylindrical melanosomes in zebrafish, which in turn is required for melanosome movement into the apical processes and maintenance of photoreceptor integrity^52^. Mutations in PMEL have previously been shown to regulate hypopigmented phenotypes in many vertebrate animals, such as in mice^53^, chickens^54^ and dogs^55^. Lymphoid enhancer-binding factor 1 (LEF 1) is a member of the LEF/T-cell-specific factor (TCF) family of the high mobility group domain transcription factors, and it is a downstream nuclear Wnt signalling pathway mediator^56^. Wnt signalling plays an important role in melanocyte development and differentiation and suppresses melanin synthesis and TYR and MITF expression^57^. In our study, the PMEL and LEF1 genes were significantly up-regulated in the skin of mink with white coats. The results of our RNA-seq analysis and subsequent qRT-PCR validation were consistent. In a more recent paper, TYR showed higher expression in the skin of black-coated sheep versus white-coated sheep, which is similar to results observed in chickens. However, in our study, the expression of TYR was not significantly different between the two coat colours. Although the results of our RNA-seq analysis and subsequent qRT-PCR validation were consistent, these results require additional research. TYR may play an important role in the pigmentation of mink hair; thus, further investigations are required to confirm the mechanism of TYR.

## Materials and Methods

### Ethics statement

All animals were handled in strict accordance with good animal practices as defined by the national and/or local animal welfare bodies. Animal experiments were approved by the Institutional Animal Care and Use Committee at the Institute of Special Animal and Plant Science, Chinese Academy of Agricultural Sciences, Changchun, China and performed in accordance with animal ethical guidelines and approved protocols.

### Mink skin sampling and total RNA extraction

In mid-May, six healthy 2-year-old male minks with black or white coat colour (3 minks per coat colour) were selected for sample collection from the fur farming Institute of Special Economic Animal and Plant Sciences, Chinese Academy of Agricultural Sciences. Two pieces of skin (1.0 cm in diameter) from the back were collected via a punch skin biopsy under local anaesthesia and were immediately placed in liquid nitrogen. Total RNA from the skin sample was extracted using the TRIzol reagent (TaKaRa, China) according to the manufacturer’s instructions. Agarose gel (1.0%) electrophoresis was used to detect RNA degradation and contamination. RNA purity was checked using a Nano Photometer^®^ spectrophotometer (IMPLEN, CA, USA), and the RNA concentration was measured using a Qubit^®^ RNA Assay Kit with a Qubit^®^ 2.0 Fluorometer (Life Technologies, CA, USA). The RNA integrity was assessed using an RNA Nano 6000 Assay Kit on an Agilent Bioanalyzer 2100 (Agilent Technologies, CA, USA). RNA samples with RIN values above 8.0 and OD 260/280 ratios between 1.8–2.0 were selected for deep sequencing.

### cDNA library construction and Illumina deep sequencing

A total of 3.0 μg RNA per skin sample per mink was used as the input material for the RNA sample preparations. Sequencing libraries were generated using the NEBNext^®^ Ultra™ RNA Library Prep Kit for Illumina^®^ (NEB, USA) following the manufacturer’s recommendations, and index codes were added to attribute sequences to each sample. Briefly, mRNA was purified from total RNA using poly-T oligo-attached magnetic beads. Fragmentation was conducted using divalent cations under elevated temperature in NEBNext First Strand Synthesis Reaction Buffer (5x). First-strand cDNA was synthesized using random hexamer primers and M-MuLV Reverse Transcriptase (RNaseH^−^). Second strand cDNA synthesis was subsequently performed using DNA Polymerase I and RNase H. Remaining overhangs were converted into blunt ends via exonuclease/polymerase reactions. After adenylation of the 3′ ends of DNA fragments, NEBNext Adaptors with hairpin loop structures were ligated to prepare for hybridization. To preferentially select 150–200 bp cDNA fragments, the library fragments were purified with the AMPure XP system (Beckman Coulter, Beverly, USA). Then, 3 μl USER Enzyme (NEB, USA) was used with size-selected, adaptor-ligated cDNA at 37 °C for 15 min followed by 5 min at 95 °C before PCR. The PCR assay was then performed with Phusion High-Fidelity DNA polymerase, Universal PCR primers and an Indexed (X) Primer. Finally, the PCR products were purified (AMPure XP system), and the library quality was assessed on an Agilent Bioanalyzer 2100. The library preparations were sequenced on an Illumina HiSeq. 2500 platform with 100 bp paired-end reads by Novogene Bioinformatics Technology Co., Ltd. (Beijing, China).

### Quality control

Raw data (raw reads) in fastq format were first processed through in-house Perl scripts. In this step, clean data (clean reads) were obtained by removing low-quality reads and those containing adapters or poly-Ns. At the same time, Q20, Q30, GC-content and sequence duplication levels of the clean data were calculated. All downstream analyses were based on clean data with high quality. Trinity (v2012-10-05) was used for assembly^58^, in which clean reads of different isoforms derived from one gene were assembled into distinct transcripts but with the same subcomponent (which can be regarded as a gene), and the longest transcript of each subcomponent was defined as the ‘unigene’ for functional annotation.

### Transcriptome annotation of unigenes

For functional annotation, all assembled unigenes that might putatively encode proteins were searched against the Nr (http://www.ncbi.nlm.nih.gov/), Swiss-Prot (a manually annotated and reviewed protein sequence database, http://www.expasy.ch/sprot/), KEGG (http://www.genome.jp/kegg/) and COG (http://www.ncbi.nlm.nih.gov/cog/) databases using the BLASTX algorithm. A typical cut-off value of E-value < 1e-5 was used. With Nr annotations, the Blast2GO program^59^ was used to assign GO (Gene Ontology) annotations to the unigenes according to the component function, biological process and cellular component ontologies. After obtaining GO annotations for all unigenes, the software WEGO^60^ was used to assign GO functional classifications to all unigenes and to understand the distribution of gene functions for the species at the macro level. Predictions of possible functional classifications and molecular pathway assignments were performed by sequence searches against the KEGG database^61^ using the BLASTX algorithm with an E-value threshold of 10^−5^.

### Differential expression, cluster analysis and Gene Ontology (GO) enrichment analysis

Different expression analysis of the two samples with different coat colours was performed using the DESeq R package (1.10.1). P values were adjusted using the Q value^62^. Genes found by DESeq with an adjusted P-value < 0.05 were assigned as differentially expressed. The identified DEGs were used for GO and KO enrichment analyses performed using the GOseq R package, which is based on the Wallenius non-central hypergeometric distribution^63^; thus, it can adjust for gene length bias in DEGs.

### Validation of DEGs by qRT-PCR

To validate our Illumina sequencing data, eight DEGs involved in the melanogenesis pathway (KITLG, LEF1, DCT, TYRP1, PMEL, Myo5a, Rab27a and SLC7A11) that were identified by the above method were randomly chosen for quantitative RT-PCR analysis using the same RNA samples as for the transcriptome profiling. In all cases, the primers were designed for qRT-PCR using the Primer Express 3.0 that spanned exon-exon boundaries and the assembled β-actin unigene (c94862_g2) was selected as an internal control. qRT-PCR was performed using the SYBR select Master Mix kit (ABI, Life, America) on an ABI 7500 Real-Time System (Applied Biosystems, USA), with the first-strand cDNA serving as the template. The qRT-PCR assay was performed in 20.0 µL reaction mixtures containing 12.5 µL 2 x SYBR select Master Mix (ABI, Life, USA), 1.0 µL of cDNA template, 1.0 µL of each corresponding forward and reverse primer for the gene of interest, and 4.5 µL PCR-grade water. The reaction was performed using the following conditions: 50 °C for 2 min and 95 °C for 30 s, followed by 40 cycles of 95 °C for 15 s and 62 °C for 1 min, with a final extension at 72 °C for 5 min. All reactions were performed with three technical replicates using one biological sample and included negative controls with no template. The relative amount of mRNA expression of each gene (expressed as adjusted Ct value) was analysed using the Stratagene Iq5 system. Adjusted cycle threshold (C(t)) values were calculated using the following equation: Ct value = 2^−ΔΔCt^ ^64^. Differences in mRNA abundance for the genes were determined by an analysis of variance, which was performed with SPSS version 19.0. Differences were considered significant at *P* values < 0.05.

Descriptions of the genes mentioned above are shown in Supplementary Table S1.

## Electronic supplementary material

SREP-17-09813-S1
