# Supplementary material for: Comparative Transcriptome Analysis of Mink (Neovison vison) Skin Reveals the Key Genes Involved in the Melanogenesis of Black and White Coat Colour
Source: Sci Rep. 2017 Sep 29;7:12461. doi: 10.1038/s41598-017-12754-0 (PMC5622100; doi:10.1038/s41598-017-12754-0)
Supplement: Supplementary file 1 — SREP-17-09813-S1 [file 41598_2017_12754_MOESM1_ESM.doc]

**Comparative Transcriptome Analysis of Mink (*Neovison vison)* Skin Reveals the Key Genes Involved in the Melanogenesis of Black and White Coat Colour**

Xingchao Song, Chao Xu, Zongyue Liu, Zhigang Yue, Linling Liu, Tongao Yang, Bo Cong, Fuhe Yang^*^

Key Laboratory of Special Economic Animal Genetic Breeding and Reproduction, Ministry of Agriculture, State Key Laboratory for Molecular Biology of Special Economic Animals, Institute of Special Economic Animal and Plant Sciences, Chinese Academy of Agricultural Sciences, Changchun 130112, China

^*^Corresponding author

**Professor** Fuhe Yang

Institute of Special Economic Animal and Plant Sciences, Chinese Academy of Agricultural Sciences.

No.4899 Juye Street, Jingyue Economic Development District, Changchun City, Jilin Province, China

[yangfuhecaas@126.com](mailto:yangfuhecaas@126.com)

Supplemental Table 1 Primers used for quantitative real time PCR. List of primers for 9 genes used in quantitative real time PCR analysis to verify differential expression of genes identified by RNA-Seq analysis

| ID number | Gene name | Primer sequence (5´→3´) | Tm (℃) | Product length (bp) |
| --- | --- | --- | --- | --- |
| c87266_g2 | KITLG | F：GTAATAGGAAGGCCGCAAACC | 59.5 | 100 |
|  |  | R：CCAAAAGCAAACCCAATTACAAG |  |  |
| c89232_g1 | LEF1 | F：GCCAGACAAGCGCAAACCT | 60.0 | 70 |
|  |  | R：GCTTTCCGTCATCAGGGTGTT |  |  |
| c85800_g1 | DCT | F：CCGGGTCACCCTCTGTAATG | 61.5 | 80 |
|  |  | R：TTGGCAATTTTGCACTGTTCAG |  |  |
| c82939_g2 | TYRP1 | F：GTTCAATGGCCAAGTCGGAAT | 58.5 | 90 |
|  |  | R：TGCGAAAATGACTGCAACCA |  |  |
| c82046_g1 | PMEL | F：TGGATGGAGGGAACAAGCA | 59.2 | 90 |
|  |  | R：CAGCCCCGGACAAATAACC |  |  |
| c92365_g1 | Myo5a | F：GGCCAGAGATGAACGAAATCA | 60.5 | 110 |
|  |  | R：CAGAACCACTCACGGTTGCA |  |  |
| c84818_g1 | Rab27a | F：ACCTGATAATGAAGCGAATGGAA | 59.0 | 90 |
|  |  | R：GATCTCTAGAGGTGTGGCCATTG |  |  |
| c79675_g1 | SLC7A11 | F：TCAAGGTGCCGCTGTTCAT | 59.6 | 80 |
|  |  | R：TGGGTCCGAATACAGGGAA |  |  |
| c94862_g2 | β-actin | F：GCGTGACATCAAGGAAGAAGC | 57.5 | 108 |
|  |  | R：CCGTCGGGTAGTTCGTAGCT |  |  |
